# The DELIVER study; the impact of research capacity building on research, education, and practice in Dutch midwifery

**DOI:** 10.1371/journal.pone.0287834

**Published:** 2023-10-31

**Authors:** Evelien Spelten, Janneke Gitsels, Corine Verhoeven, Eileen K. Hutton, Linda Martin

**Affiliations:** 1 Violet Marshman Centre for Rural Health Research, Rural Health School, La Trobe University, Melbourne, Australia; 2 Amsterdam UMC location Vrije Universiteit Amsterdam, Midwifery Science, Amsterdam, the Netherlands; 3 Midwifery Academy Amsterdam Groningen, InHolland, Amsterdam, the Netherlands; 4 Department of Midwifery, School of Health Sciences, University of Nottingham, Nottingham, United Kingdom; 5 Department of Obstetrics and Gynaecology, Maxima Medical Centre, Veldhoven, the Netherlands; 6 McMaster Midwifery Research Unit, McMaster University Hamilton, Canada; Muhimbili University of Health and Allied Sciences (MUHAS), SWEDEN

## Abstract

**Background:**

Few examples exist of research capacity building (RCB) in midwifery. As in other jurisdictions, at the turn of this century midwives in the Netherlands lagged in research-based practice. Dutch professional and academic organisations recognised the need to proactively undertake RCB. This paper describes how a large national research project, the DELIVER study, contributed to RCB in Dutch midwifery.

**Methods:**

Applying Cooke’s framework for RCB, we analysed the impact of the DELIVER study on RCB in midwifery with a document analysis comprising the following documents: annual reports on research output, websites of national organizations that might have implemented research findings, National Institute for Public Health and the Environment (RIVM)), midwifery guidelines concerning DELIVER research topics, publicly available career information of the PhD students and a google search using the main research topic and name of the researcher to look for articles in public papers.

**Results:**

The study provided an extensive database with nationally representative data on the quality and provision of midwifery-led care in the Netherlands. The DELIVER study resulted in 10 completed PhD projects and over 60 publications. Through close collaboration the study had direct impact on education of the next generation of primary, midwifery care practices and governmental and professional bodies.

**Discussion:**

The DELIVER study was intended to boost the research profile of primary care midwifery. This reflection on the research capacity building components of the study shows that the study also impacted on education, policy, and the midwifery profession. As such the study shows that this investment in RCB has had a profound positive impact on primary care midwifery in the Netherlands.

## Introduction

In the Netherlands midwives are autonomous, medical health care practitioners who approach pregnancy and birth care from a physiological, rather than a pathological perspective [1] and have a solid reputation for delivering high quality midwife-led perinatal care. In 2019, 87% of pregnant women started their care with a midwife [2]. Despite providing care to the majority of Dutch women, all metrics of research productivity indicated that midwife-led research was largely absent prior to 2010. Thus, Dutch midwives had a limited scientific body of knowledge about critical aspects of care and evidence-based care. A lack of insight into mechanisms explaining benchmark data, such as differences in the number of medical interventions between both primary and secondary maternity care facilities, to reflect on care provision existed. Very few midwives had a postgraduate degree or would consider research as a career option. Acknowledging this hiatus, a national movement started around 2010 to invest in research capacity building (RCB) [3].

In recognition of the importance of evidence-based practice, The World Health Organisation’s (2009) [4] global standards on the education of midwives, stressed the importance of research capacity building (RCB). RCB has been defined as ’a process of individual and institutional development which leads to higher levels of skills and greater ability to perform proper research [5]. RCB programs use a variety of approaches with the most prominent examples being fellowships and internships. Programs focus on early career researchers as well as on experienced clinicians through adjunct or conjoint to promote clinical academic career paths [6, 7]. However, it is well recognised that successful RCB includes provision of, for example, a supportive research climate and infrastructure, enhancing collaborations and network building. In this context, McAllister [8] and Matus et al. [9], identified the importance of a shared rather than a top-down approach to working with novice researchers and Wenger [10] and Trostle [5] emphasised the need for flexibility and broadness in supervision. Matus et al. [9] mentioned the importance of valuing research for excellence and supporting clinicians in practice. McAllister [8] considered a community of practice as a tried and tested model. Good mentorship and knowledge exchange contribute to success of RCB, as well as interdisciplinary collaboration which requires supportive network environments, and community-based research [5, 8–11].

Brew et al., [12–16] stressed the benefits of integrating *education* with RCB as this offers students a different professional perspective. Research enhanced education should start in undergraduate degrees and not keep students at arm’s length [13, 17]. Green et al. [17] reported on a case study of developing nursing and midwifery RCB as part of education moving into a university setting. The RCB developed from an initial all-inclusive approach into more leading-edge research. There was a strong convergence of teaching and research which was supported by academic leadership and educational management working in tandem [17].

Crucial to the *success* of RCB is the development of critical mass and allowing time for the development and embedding of research. Cooke recommends at least a 10-year timeframe before considering the actual impact of RCB efforts [18]. Time is needed to develop an academic identity in what Ennals summarises as "doing, being, becoming and belonging" [19]. Protected time, especially in a teaching environment, is singled out as one of the critical factors for RCB success and thus also failure [20]. Closely related to protected time is attention to academic writing and critical thinking, skills that take and need time to develop [11, 21]. Women experience inequity in academia and in obtaining grant funding and these challenges are intensified in nursing and midwifery where research domains are in just evolving [7, 11]. RCB should benefit education, clinical practice and the wider society and result in research that values engagement and impact with local and global issues [21, 22]. In an evaluation of a graduate midwifery research intern program Hauck et al. [7] recognised the relevance of RCB not just for clinical practice but for health care in general, through for example, contributions to policy development. Carrick-Sen et al. [6] mentioned the importance of research that improves patient outcomes, RCB is an important mechanism to improve health service delivery [6, 23]. Brew related successful RCB to the reciprocal influence of political, institutional and disciplinary factors, stating that "today’s society demands creativity and the ability to deal with complexity and uncertainty, change is endemic” [13].

Around 2010, in the Netherlands, several initiatives involving the three Midwifery Academies as well as the professional bodies of midwives and obstetricians were taken to improve RCB in midwifery. One of the initiatives was the establishment of the first university-based research department of Midwifery Science [3]. At its inception, the department, together with the teaching-based Midwifery Academy Amsterdam Groningen (AVAG), undertook the DELIVER Study (Data Eerste LIjns VERloskunde, a Dutch acronym for evidence on midwifery care), see Box 1. The aim of the DELIVER study was to contribute to evidence-based practice in primary care midwifery and to research capacity building. With the data collection for the DELIVER study ending approximately ten years ago, now seems a good time to evaluate and reflect [18]. While the primary intention of the DELIVER project was to increase research capacity and academic output, after ten years it is equally interesting to reflect on the impact the study had beyond research. The research question for this paper therefore was: how did the DELIVER study contribute to RCB in Dutch midwifery? In addition to academic output, the focus is on subsequent impact on education, midwifery practice, and the profession.

**BOX 1: The DELIVER study: data collection and research capacity building**

The design of the DELIVER study is described in more detail elsewhere [80, 81]. Data for the DELIVER study were collected between September 2009 and April 2011. Clients and their partners, midwives and other healthcare professionals across the Netherlands participated in the study. As a prospective cohort study, clients from twenty midwifery practices completed up to three questionnaires during their pregnancy. The client data were linked to data from the Netherlands Perinatal Register and electronic client records kept by midwives. Midwives and practice assistants from the twenty participating practices completed a questionnaire and recorded work-related activities in a diary for one week, to assess workload. Data at client, midwife and practice level were linked. Another questionnaire on the organisation of midwifery-led care was sent to all, ca. 500, Dutch midwifery practices. Additionally, partners of pregnant women and other care providers were asked about their expectations and experiences regarding the care delivered by midwives and in six practices client consults were videotaped to objectively assess daily practice.

In total, 7685 clients completed at least one questionnaire, 136 midwives and assistants completed a diary with work-related activities (response 100%), 99 midwives completed a questionnaire (92%), 30 partners of clients participated in focus groups, 21 other care providers were interviewed, 305 consults at six midwifery practices were videotaped and 319 practices across the country completed a questionnaire (61%). This extensive database lay at the heart of a 10 PhD projects, a myriad of papers and conference presentations, and impacted practice, education, xand policy.

## Methods

The six principles developed by Cooke [18, 24] to evaluate RCB are [1] build skills and confidence, [2] support research that is close to practice, [3] identify linkages, collaborations and partnerships, [4] ensure appropriate dissemination and impact, [5] include elements of continuity and sustainability and [6] establish an appropriate infrastructure. The principles can be applied at an individual, team, organisational and supra-organisational level. Cooke [25] noted that measures of traditional academic outputs (such as peer reviewed publications) do not provide a complete picture, and recommends that assessment also consider social impact (e.g. health improvements), related to what we now call the engagement and impact of academic work. Using Cooke’s framework [18, 20, 24] and its application, we analysed the impact of the DELIVER study on RCB in midwifery with a document analysis comprising the following documents: annual reports on research output, websites of national organizations that might have implemented research findings (e.g. Royal Dutch Organization of Midwives (KNOV), National Institute for Public Health and the Environment (RIVM)), midwifery guidelines concerning DELIVER research topics, publicly available career information of the PhD students and a google search using the main research topic and name of the researcher to look for articles in public papers. The CVs were not publicly available, but consent was acquired prior to accessing. The outputs of the study described in the document content were coded and categorised in line with the framework’s six principles.

The design of the DELIVER study is described in more detail elsewhere [26, 27]. Data for the DELIVER study were collected between September 2009 and April 2011. Clients and their partners, midwives and other healthcare professionals across the Netherlands participated in the study. As a prospective cohort study, clients from 20 midwifery practices completed up to three questionnaires during their pregnancy. The client data were linked to data from the Netherlands Perinatal Register and electronic client records kept by midwives. Midwives and practice assistants from the twenty participating practices completed a questionnaire and recorded work-related activities in a diary for one week, to assess workload. Data at client, midwife and practice level were linked. Another questionnaire on the organisation of midwifery care was sent to all, approximately 500, Dutch midwifery practices. Additionally, partners of pregnant women and other care providers were asked about their expectations and experiences regarding the care delivered by midwives. As a final part of the DELIVER study data collection, in six practices client intake consults were videotaped to objectively assess daily practice.

In total, 7685 clients completed at least one questionnaire, 136 midwives and assistants completed a diary with work-related activities (response rate 100%), 99 midwives completed a questionnaire (response rate 92%), 30 partners of clients participated in focus groups, 21 other care providers were interviewed, 305 consults at six midwifery practices were videotaped and of the 523 practices in the country, 319 completed a questionnaire (response rate 61%). This comprised the total data set for the DELIVER study. PhD candidates were able to consider all collected data: surveys, focus groups, video recordings, depending on the relevance for their project.

The authors of this paper were actively involved with the DELIVER project and the initiatives to advance the evidence base and the professional status of midwifery. They have used their own experiences with the project to add to the narrative of this paper. ES is a psychologist and was Chief Investigator on the project and co-supervisor; JG is a midwife, she runs one of the participating practices and was one of the PhD candidates; CV is a midwife who joined the department of Midwifery Science after her PhD in 2013 where she currently leads a second research program as a follow-up to/continuation of the DELIVER study; EH is a midwife, head of a midwifery department and first Chair of the midwifery science department and principal supervisor; LM is a psychologist, a lecturer at the midwifery Academy and was one of the PhD candidates.

### Ethics statement

This study is a reflection on the process of the DELIVER study and does not involve human participants, therefore no ethics approval was needed. The design and conduct of the DELIVER study were approved by the Medical Ethics Committee of the VU University Medical Centre Amsterdam.

## Results

Using Cooke’s framework [18, 20, 24], we provide a description of how the RCB process was implemented followed by an account of direct research and academic output, then a summary of the impact on education and on the profession (primary through policy and practice). Findings are summarised in several tables: Table 1 provides a summary of the six principles in relation to our work. Table 2 provides a summary of the 10 PhD projects based on the DELIVER study and the related research output [26–71], which were the core research part of the study. In Table 3 the network activities related to the PhD are summarised, and finally Table 4 captures the dissemination and impact of the study from 2010–2020.

**Table 1:** Research capacity building model, adapted from Cooke and complemented.

RCB Objective	Outcome Indicator
1. Build skills and confidence	• Skills developed • Evidence of career development • Evidence of confidence-building; building and sharing new skills with others • Availability and use of training funds • Research undertaken, funding approved • Working with other professional groups in research • Responses to needs based work • Evidence of secondment opportunities offered and taken up
2. Close to practice	• Evidence of supported projects related to issues/questions identified in clinical practice • Evidence of patient-centred outcome measures in projects • Examples of critical thinking used in practice • Evidence of research questions being developed with practice, needs and priorities • Co-ordination of research programmes between health organisations and university • Action research orientated approaches undertaken • Evidence on level and nature of service user involvement • Evidence of supporting service user links in research • Development of service user panels
3. Linkages, collaborations and partnerships	• Evidence of new partnerships • Links with universities • Network development • Inter-site/inter-speciality research collaboration • Work with funding bodies
4. Appropriate dissemination and impact	• Local dissemination • Conference presentations • Papers in research and practice journals • Dissertation • Seminar programs relating to research undertaken • Dissimination of information for pregnant women and their loved ones. • Examples of evidence-based practice and applying locally developed knowledge in strategy policy and practice
5. Continuity and sustainability	• Access to funding to continue education of skills • Examples of continued collaboration • Long-term plans developed for a project • Continued commitment to working in research • Building and continuation of Childbirth Network
6. Infrastructure	• Evidence of project management in projects • Description of mentorship and supervision structures • Evidence of sustained collaboration • Links with management established • Evidence of strategic future planning • Protected time made available • Network of academic midwifery practices

**Table 2:** Summary of individual projects supported by the DELIVER-study (2010–2020).

ID	Grade/profession	Research topic	Skills development	Dissemination	Publication	Professional continuity and sustainability
1	Health Scientist	The role of clients, midwives and health policy in preventing infectious diseases during pregnancy	Academic writing Communication and presentation skills. Quantitative and qualitative research Data collection Data analyses Video-analysis Collaboration with other researchers	5 international papers Oral and poster presentations RIVM^1^	Pereboom et al. (2013; 2014a; 2014b; 2014c; 2014d)	Senior-researcher
2	Psychologist, lecturer	Counselling for prenatal anomaly screening: parents and midwives perspectives and client-midwife communication	Academic writing Communication and presentation skills. Quantitative and qualitative research Data collection Data analyses Video-analysis Collaboration with other researchers	10 international papers Oral and poster presentations RIVM^2^ AVAG^3^	Martin et al. (2013; 2014; 2015a; 2015b; 2016) Gitsels–van der Wal et al. (2015a; 2015b) Spelten et al. (2015) Pereboom et al. (2014) Baron et al. (2017)	Post-doc Senior-researcher Lecturer
3	Midwife, theologian	Religious beliefs in decision-making and counselling around prenatal anomaly screening	Academic writing Communication and presentation skills. Quantitative and qualitative research Data collection Data analyses Video-analysis Collaboration with other researchers	12 international papers Oral and poster presentations RIVM^2^ AVAG^3^	Gitsels et al. (2014a; 2014b; 2014c; 2015a; 2015b) Martin et al. (2014; 2015a; 2015b; 2016) Spelten et al. (2015) Pereboom et al. (2014) Baron et al. (2017)	Post-doc Senior-researcher
4	Lecturer, midwife	On the use and determinants of prenatal healthcare services	Academic writing Communication and presentation skills. Quantitative research Data analyses Systematic review Collaboration with other researchers	5 international papers Oral and poster presentations	Feijen–de Jong et al. (2012; 2013; 2015a; 2015b) Boerleider et al. (2015)	Senior-researcher lecturer
5	Medical doctor	Non-Western women in maternity care in the Netherlands	Academic writing Communication and presentation skills. Quantitative and qualitative research Data collection Data analyses Collaboration with other researchers	6 international papers Oral and poster presentations	Boerleider et al. (2013a; 2013b; 2013c; 2014; 2015) Feijen–de Jong et al. (2015)	Lecturer
6	Lecturer, midwife	Management of labour pain in midwifery care	Academic writing Communication and presentation skills. Quantitative and qualitative research Data collection Data analyses Collaboration with other researchers Cochrane systematic review	5 international papers Oral and poster presentations KNOV^4^	Klomp et al. (2012; 2013; 2014; 2016); Manniën et al. (2012)	Post-doc Lecturer
7	Psychologist, lecturer	The organization of midwifery care in the Netherlands	Academic writing Communication and presentation skills. Quantitative and qualitative research Data collection Data analyses Collaboration with other researchers	5 international papers Oral and poster presentations	Warmelink et al. (2015a; 2015b; 2017a; 2017b); Wiegers et al. (2014);	Post-doc Lecturer
8	Health scientist	Maternal health and prenatal health education in midwife-led primary care	Academic writing Communication and presentation skills. Quantitative research Data collection Data analyses Video analyses Collaboration with other researchers	4 international papers Oral and poster presentations	Baron et al. (2013; 2015; 2017a; 2017b)	Senior researcher
9	Midwife, lecturer	Continuous support for women during childbirth	Academic writing Communication and presentation skills. Quantitative and qualitative research Data analyses Collaboration with other researchers	3 international papers Oral and poster presentations	Baas et al. (2015; 2017a; 2017b)	Lecturer
10	Health scientist	Pregnancy related anxiety	Academic writing Communication and presentation skills. Quantitative research Data analyses Collaboration with other researchers	4 international papers Oral and poster presentations	Westerneng et al. (2015; 2017; 2019; 2022)	Lecturer

RIVM: the National Institute for Public Health and the Environment from the Ministry of Health, Welfare and Sport

^1^ https://www.rivm.nl/weblog/rol-van-clienten-verloskundigen-en-gezondheidszorgbeleid-bij-voorkomen-van-infectieziekten

^2^ https://www.pns.nl/sites/default/files/2021-03/210224%20Factsheet%20Counseling%3B%20gespreksleidraad.pdf

AVAG: AVAG Midwifery Academy Amsterdam Groningen

^3^ https://www.verloskunde-academie.nl/counseling-prenatale-screening/; https://www.pns.nl/professionals/nipt-seo/scholing-counselors/opleiding

KNOV: Royal Dutch Organization of Midwives

^4^ https://assets.knov.nl/p/557056/none/PDF%20Vakkennis/Standpunt_Voorlichting_over_pijn_en_pijnbehandelingen_tijdens_de_baring.pdf

**Table 3:** Summary of research network by the PhD-projects based on the DELIVER-study.

ID	Research group/network	Support provided or research outputs	Group status
101	NIVEL	Training in video-observation software and methods (RIAS); On the job training questionnaire development and validation Scientific feedback meetings	Closed
102	BoL	Theological analyses of interview data	Ongoing
103	TRIDENT-1 AND TRIDENT-2	Participation in national studies of prenatal anomaly screening	Ongoing
104	RIVM	Implementation of research output	Ongoing
105	Childbirth Network	Clientparticipation	Ongoing
106	Groningen UMC / Amsterdam UMC/AVAG	Supervision PhD’s	Ongoing
107	International midwifery education: McMaster University; Karolinska Institute	Internships for students	Ongoing
108	MRNN	Coordination midwifery research projects	Ongoing
109	KNOV	Consultation as experts for guidelines development	Ongoing
110	AVAG	Support PhD projects Bachelor and master’s fellowships	Ongoing

**NIVEL:** Netherlands Institute for Health Services Research; **BoL:** Beginnings of life–multidisciplinary research group (Free University, department Theology and Religious studies, department Midwifery Science/ department Community Genetics /AVAG); **VSOP:** Patient Alliance for Rare and Genetic Diseases; **RIVM:** National Institute for Public Health and the Environment; **MRNN:** Midwifery Research Network the Netherlands; **KNOV:** Royal Dutch Organization of Midwives; **AVAG:** Midwifery Academy Amsterdam Groningen; **UMC:** University Medical Center.

**Table 4:** Summary of dissemination and impact of the DELIVER-study (2010–2020).

Outputs	Details
*PUBLICATIONS*	
Published international peer-reviewed papers	43
National journals	Summary of PhD projects, linkage to national issues, policies, and professional journals.
*DISSEMINATION*	
Media	Press releases, radio, news papers, popular press
Organisation of national conferences and symposia	Symposium at the end of the professoriate of Professor Hutton National DELIVER results symposium Midwifery School Lustrum Symposium
National midwifery conferences	Knowledge Portal Midwifery, KNOV, Regionale Center Prenatal anomaly screening
International conferences	ICCH, ISPD, ICM, and other midwifery conferences
*AWARDS AND RECOGNITION*	
Research award	Wij Inholland award (2016)
*TEACHING AND TRAINING*	
Mirror/reflection meetings following video-recordings	Video-feedback communication training
Workshops	Workshop Shared Decision Making KNOV (2013) Knowledge Portal Midwifery (2013–2016)
Writing workshops and retreats	Annually
PhD-candidate days to improve skills etc	Twice a year
Evidence of career progression as a result	10 timely completed PhDs
Policy making	RIVM (2015 untill current)
Appointment of first Dutch full Professor in midwifery	AmsterdamUMC, department Midwifery Science (2019)

**KNOV:** Royal Dutch Organization of Midwives; **ICCH:** International Conference on Communication in Healthcare; **ISPD:** International Society for Prenatal Diagnosis; **ICM:** International Confederation of Midwives; **RIVM:** the National Institute for Public Health and the Environment from the Ministry of Health, Welfare and Sport

### Academic and research outcomes

The DELIVER study provided midwives and other health care professionals such as health scientists and psychologists the opportunity to *build research skills and confidence* (See Table 1) by undertaking a PhD on a midwifery related topic (Table 2). All PhD theses were publication based, thereby developing academic publishing skills, and building the body of research to inform practice. PhD candidates participated in regular full day seminars led by Midwifery scientists to provide *infrastructure* and built a community of practice among novice researchers. Seminar topics included responding to reviewer comments, developing effective conference presentations (oral or poster), specific study methods and approaches to analyses. Participants were encouraged to bring challenges they were encountering to the group for discussion with peers, thus *building on the skill set and confidence* of the researchers. Since PhD students did not all start at the same time more experienced students were able to mentor novices thus moving into a supervisor-student role and *building confidence* throughout the process on different levels. In addition, each PhD candidate needed to attain 30 European Credits from trainings programs tailored to individual competencies and learning objectives as well to more general requirements, such as a scientific integrity course. The PhD candidates independently selected their research topic, which contributed to their motivation and completion. They used the DELIVER data set augmented with additional data collection if needed. The project team of the DELIVER study supervised PhD candidates to complete their thesis work according to university standards and navigated additional research training and research time needs as necessary, thus *establishing an appropriate infrastructure*. Both the midwifery academy and the midwifery practice provided *protected time* for the lecturers and the practising midwife to work on their PhD to ensure timely completion. The midwifery academy Amsterdam Groningen (AVAG) funded nine projects; the Royal Dutch Organisation for Midwives (KNOV) funded the tenth project (project 3). The supervisory teams of 8 of the 10 candidates included professors from other organizations, such as other, non-Dutch, universities, or health services research institutes (projects 2–7, 9, 10). This provided the PhD candidates with *linkages* to professionals to facilitate their research careers within and outside midwifery science.

As part of the DELIVER project, an interdisciplinary advisory panel including midwives, obstetricians, medical doctors, health scientists and psychologists enhanced *networking*, *linkages*, *and collaboration*. The design of the DELIVER study was modelled on the Dutch National Study on Diseases and Interventions in General Practice using, where relevant, the same set of validated questionnaires, which added to the validity of the study. Additional questions, for example about pain management in labour and prenatal anomaly screening, were added based on *specific midwifery context*, *aligning with Cooke’s notion of the research being close to practice* [33–37, 50–53].

Table 2 identified the 10 PhD projects that were part of the DELIVER project, as well as described the career development of the PhD candidates. An important outcome was that all 10 PhD candidates completed their PhD program; three of them while working as a lecturer at the Midwifery Academy Amsterdam Groningen, one combined her PhD project with working in a midwifery practice; three candidates worked full-time on their project, and two worked full-time as a researcher on both the DELIVER-study as well as their PhD project. One PhD-candidate was a DELIVER study project leader, she co-authored over 10 DELIVER papers (project 6). Four PhD candidates used the video-recordings as part of their project (Table 2: video analysis). Additionally, the DELIVER study facilitated four established researchers in their research careers by publishing manuscripts in peer-reviewed journals (n = 7) and attending (international) conferences [3, 26, 27, 72–75]. As summarised in Table 2, the DELIVER study’s 10 PhD projects contributed significantly to the scientific output of the study, with a total of 43 peer-reviewed internationally published papers, thus ensuring *appropriate dissemination and impact*. Furthermore, the DELIVER studies resulted in ongoing robust collaboration network that for instance facilitates new research projects and dissemination of research findings (Table 3). And finally, the results of the different PhD-projects of the DELIVER study were disseminated in national professional journals, national media, and national conferences and international conferences.

Five out of ten PhD graduates received a post-doc position (projects 2–4,6,7); of whom one received a postdoctoral fellowship of the professional association (project 3). Two individuals became researchers at other universities, one in the Netherlands and one in another country (projects 1,5). Four combined post-doc positions with a role as a lecturer while one combined the post-doc position with practising midwifery. Two researchers did not continue in research; however, they were able to disseminate the results of their projects in education in their function as lecturers in the midwifery academies (projects 8,10). See also table 2.

### Education

The DELIVER study was an initiative of one of the Midwifery Academies, under the auspices of the Professional Midwifery Education Foundation.

The Midwifery Academy Amsterdam Groningen (AVAG) initiated the study, and five of their lecturers (comprising 14% of all lecturers) undertook PhD study (projects 2,4,6,7,9) which provide excellent opportunities for *interaction with practice* as well as with education. These lecturers demonstrated to both colleagues and students the relevance of research and evidence-based working and verified that research could be a career option for midwives. Students were involved with explorative analyses during their research internships, and some participated in the DELIVER study as research assistants, e.g., through transcription of interviews, supporting symposia and workshops. Published papers were discussed in Journal clubs and students participated in research meetings. To ensure *continuity* for the DELIVER study, the Midwifery Academy invested in a program of Masters-funding to encourage and support midwives to undertake a master’s degree [30].

#### Midwifery practice

Midwives of 20 midwifery practices, a representative sample of all practices in the Netherlands participated in the study, thus *supporting research that is close to practice* [26]. These midwives advised the research team about relevant research topics and during the study they were engaged to provide feedback on research findings to guarantee that the research findings were interpreted using a practice lens. The selection of PhD topic was related to practice experience of the midwives involved, e.g., prevention of infectious diseases, pain management, the determinants of midwifery care utilization, and counselling for prenatal anomaly screening.

The collaborative approach to the research promoted *reciprocity* among participating midwives and researchers. For instance, the video recordings of consultations to discuss prenatal anomaly screening were used to enhance consultations skills among participating midwives. This method was relatively new to them. For most midwives, it was the first time they had seen themselves in action and it was the first time they received this kind of feedback on their work practice. Dissemination of findings in workshops and training, allowed for direct translation of research results into practice.

Since the introduction of the RCB intervention students have learned to include scientific evidence in practice, resulting in midwifery practices feeling comfortable seeking evidence and using an evidence-based approach. The ensuing established Masters Fund of AVAG has resulted in an increase of midwives with a master’s degree in midwifery practices and a close educational collaboration with the Masters’ Course Evidence Based Practice in Health Care (University of Amsterdam), Midwifery undergraduate students can choose to do a pre-master program there, allowing for easier access to a Masters program.

### The profession

One of the broader impacts on the profession was the building *of linkages*, *collaborations and partnerships*, which helped to *establish an appropriate infrastructure* to ensure *continuity and sustainability* of research. While these partnerships may have started as research collaboration, they impacted on both education and the profession as well. The DELIVER study and the establishment of the first Dutch University based Department of Midwifery Science (e.g., multidisciplinary academic research group on midwifery) was part of a national incentive to improve evidence-based practice for primary care midwifery. The chair for the Department was recruited from Canada, which broadened international *collaboration* (e.g. peer-reviewed papers from an international team of authors; student-exchange program) and perspective (e.g. encouragement of international cooperation in research and education).

Table 3 summarises the networks based on or arising from the PhD projects. Through the DELIVER study, nationally, the Department of Midwifery Science was embedded and linked not only to one of the midwifery academies and the university but also to the Netherlands Institute for Health Services Research (Nivel) and policy through meetings with and provision of research scholarships by the Royal Dutch Organization of Midwives (KNOV) and the National Institute for Public Health and the Environment from the Ministry of Health, Welfare and Sport (RIVM). By collaborating in the establishment of the Midwifery Research Network Netherlands [76], collaboration with the other two Dutch midwifery academies and their research initiatives was improved as well as expanded.

Furthermore, results led to policy changes such as revised guidelines around quality of care on for example pain management, prevention of infectious diseases, and counselling for prenatal anomaly screening. And as a final major milestone, the DELIVER study contributed greatly to the establishment of the first Dutch primary care midwifery chair with a Dutch midwife as a professor.

The 10 PhD projects contributed to the midwifery science department becoming a well-recognised player in both the field of midwifery research and the academic world, now with four associate professors and a full professor in Midwifery since 2019. The department has a *sustainable infrastructure* enabling effective running of research projects. This, in turn, contributes to the *sustainability* of the midwifery research department and ensures a supportive environment for new PhD students.

The results of the DELIVER study have also contributed to the establishment of the Childbirth Network [https://www.childbirthnetwork.nl/]. The aim of the Childbirth Network is to connect midwifery practice, education, and research. A website has been developed where pregnant women and midwives can find evidence-based information. Next to this, a network of academic midwifery practices has been established. Within the Childbirth Network, six new research projects have started. The scientific work is informing practice and education by establishing a body of evidence. And equally, practice informs research by collecting data and contributing important research questions. In collaboration with Midwifery Academy Maastricht the Dutch Midwifery Case Registration System (VeCaS) was established. This system includes routinely collected data from electronic primary midwifery care registration systems used by Dutch Midwifery care practices across the Netherlands, and is part of the Childbirth Network [77].

Midwives participating in the network of academic practices had the impression that more women opted for a home birth during the COVID-19 pandemic. As an example of practice-based research, researchers from the midwifery science department then decided to examine this impression by researching the course of pregnancy and birth of low-risk women who started their care in primary midwifery care, and the accompanying maternal and neonatal health outcomes differed during the first wave of the COVID-19 pandemic (2020) compared to the pre-pandemic period (2019), using the VeCaS data. The conclusion of this research project was that compared to the pre-pandemic period, during the pandemic there was indeed a higher rate of planned and actual home birth among low-risk pregnant women in the Netherlands [78].

## Discussion

### Main findings

The well-designed multicentre multidisciplinary prospective DELIVER study was a well-thought-out investment designed to boost research in midwifery-led care. The study provided an extensive database with nationally representative data on the quality and provision of midwifery-led care in the Netherlands [25, 27, 79]. The study was undertaken at a time when there was a widespread initiative to improve the academic profile of primary care midwifery with extended impact on education and midwifery practice and policy. The DELIVER study resulted in 10 completed PhD projects and over 60 academic publications. The study had a direct impact on the education of the next generation of midwives and on midwifery practice and the profession. Close collaboration with primary midwifery care practices and governmental and professional bodies, allowed for direct translation and dissemination of research results.

### Interpretation of findings

**1. Academic impact**.

The RCB impact of the DELIVER study can be highlighted at a number of levels.

The direct academic output was substantial, all PhD projects consisted of a collection of published and peer-reviewed papers, published in high-ranking academic journals. The PhD projects were an integral part of the study with the number gradually increasing from 4 to 10. Because PhD candidates independently selected their topic of research, there was wide variation in topic, study design and output.

The department of Midwifery Science has made a professional impact and is now headed by a professor of Midwifery and has become an important and influential department within the university.

**2. Impact beyond academia: education, practice and policy**.

*Education*. The Midwifery Academy inspired and initiated this RCB project. It allowed for lecturers integrating their own research into their classes, and lecturers being role models for students and colleagues. The integrated infrastructure with the new University Department of Midwifery Sciences ensured continuation and easy access to a research environment.

Students directly experienced the importance of an evidence- based approach to practice and these experiences may have provided the catalyst for their future involvement with research.

*Practice*. Twenty midwifery practices and 119 midwives were actively involved in the study. Midwives involved in the video study received feedback on their work practice as a form of continuous professional development. The practices were spread over the Netherlands which allowed for signalling of differences between regions and national outcomes, on topics such as prenatal screening uptake. The spread allowed for national exposure of practising midwives to research and contributed to the normalisation of participating in research. PhD-midwives experienced increased professional respect from colleagues and a more equal position with obstetricians for example in joint research projects, both national and internationally. In addition, there are continuing professional workshops and seminars and attendance at conferences where findings relevant to clinical practice are presented.

*Policy*. The projects contributed to policy development for example on infectious diseases, labour pain management and counselling for prenatal anomaly screening and one PhD-midwife has become an expert in the Medical Disciplinary Committee for midwifery and obstetrics conflict assessor in Midwifery Policy, as well as a member of the research committee Pregnancy and Childbirth ZonMw (The Netherlands Organisation for Health Research and Development)

Through the DELIVER study, the Department of Midwifery Science was established, the department secured a seat at relevant tables. This improved the standing of the profession and strengthened the voice of primary care midwifery.

**3. Reflecting on RCB**.

In this paper, we retrospectively looked at the impact of the DELIVER study on RCB in primary care midwifery. The Cooke framework provided an excellent structure to support our reflection, 10 years on and allowed reflection on and analysis of the impact of the DELIVER study. An obvious recommendation for future research is incorporating the full range of aspects of RCB prospectively. More traditional aspects of evaluation such as academic publications are already documented automatically through systems such as ORCID. However other RCB characteristics, often relating to the increasingly important *impact* of research, are less easily determined retrospectively and would benefit from prospective data collection.

### Limitations and strengths

A limitation of this reflection was the reliance on our own reflections which may have colored the results or left out aspects of RCB, given the time that has passed since the start of the study. For example, we may have been unaware of the impact of the DELIVER study on certain policy documents. Equally, we may have underestimated the impact of our output by following the Cooke model and not including for example analysis using SciVal or similar platforms. When considering all aspects of RCB, any future study should not only include close collaboration with practices and professionals, but should more strongly include the voice of consumers, pregnant women and their partners, in the whole research trajectory, as is being established now in the Childbirth Network. A strength of this reflection is that the authors were and are directly involved in primary care midwifery in multiple roles and witnessed the impact of this RCB process over the 10 years since the start of the study.

## Conclusion

The well-designed multicentre multidisciplinary prospective DELIVER study was a considered investment to boost research in midwifery-led care. This reflection considered this investment on all research capacity building components and demonstrated that the study also impacted on education, policy and the midwifery profession. As such the study shows that this considered investment in RCB has had a profound positive impact on positioning primary care midwifery, now and in the future.
